# Sprouty2 inhibits amphiregulin-induced down-regulation of E-cadherin and cell invasion in human ovarian cancer cells

**DOI:** 10.18632/oncotarget.13162

**Published:** 2016-11-07

**Authors:** Jung-Chien Cheng, Hsun-Ming Chang, Siyuan Xiong, Wai-Kin So, Peter C. K. Leung

**Affiliations:** ^1^ Department of Obstetrics and Gynaecology, Child & Family Research Institute, University of British Columbia, Vancouver, British Columbia, Canada V5Z 4H4

**Keywords:** amphiregulin, E-cadherin, ovarian cancer, sprouty2

## Abstract

Similar to *Drosophila* Sprouty (SPRY), mammalian SPRY proteins inhibit the receptor tyrosine kinase-mediated activation of cellular signaling pathways. SPRY2 expression levels have been shown to be down-regulated in human ovarian cancer, and patients with low SPRY2 expression have significantly poorer survival than those with high SPRY2 expression. In addition, epidermal growth factor receptor (EGFR) is overexpressed in human ovarian cancer and is associated with more aggressive clinical behavior and a poor prognosis. Amphiregulin (AREG), the most abundant EGFR ligand in ovarian cancer, binds exclusively to EGFR and stimulates ovarian cancer cell invasion by down-regulating E-cadherin expression. However, thus far, the roles of SPRY2 in AREG-regulated E-cadherin expression and cell invasion remain unclear. In the present study, we show that treatment with AREG up-regulated SPRY2 expression by activating the EGFR-mediated ERK1/2 signaling pathway in two human ovarian cancer cell lines, SKOV3 and OVCAR5. In addition, overexpression of SPRY2 attenuated the AREG-induced down-regulation of E-cadherin by inhibiting the induction of the E-cadherin transcriptional repressor, Snail. Moreover, SPRY2 overexpression attenuated AREG-stimulated cell invasion and proliferation. This study reveals that SPRY2 acts as a tumor suppressor in human ovarian cancer and illustrates the underlying mechanisms that can be used as possible targets for the development of novel therapeutics.

## INTRODUCTION

Ovarian cancer is the fifth most common cause of cancer death in women due to the lack of effective screening methods, a paucity of symptoms during the early stages of the disease, and limited responses to treatment in the late stages of disease. Many studies have demonstrated that epidermal growth factor receptor (EGFR) is overexpressed in human ovarian cancer, and EGFR overexpression is associated with more aggressive clinical behavior and a poor prognosis [1, 2]. Although multiple ligands can bind to and activate EGFR, only epidermal growth factor (EGF), amphiregulin (AREG) and transforming growth factor-α (TGF-α) bind exclusively to EGFR [3, 4]. AREG exerts its biological functions by activating EGFR in an autocrine and/or paracrine fashion [5, 6]. A recent study demonstrates that AREG is the most abundant EGFR ligand in ascites fluid collected from patients with ovarian cancer and in conditioned media from ovarian cancer cells [7]. In addition, treatment with a neutralizing monoclonal anti-AREG antibody inhibits the growth of ovarian cancer xenografts and enhances chemotherapy efficacy [7]. Taken together, these results indicate that AREG plays important roles in regulating ovarian tumorigenesis.

The majority of women with ovarian cancer are diagnosed at a late stage, when the cancer has spread beyond the confines of the ovary. E-cadherin is a transmembrane glycoprotein localized on the surface of epithelial cells in regions of cell-cell contact, known as adherens junctions. E-cadherin mediates calcium-dependent cell-cell adhesion, which is important for maintaining cell polarity and normal epithelial architecture [8]. Loss of E-cadherin expression is the hallmark of the epithelial-mesenchymal transition, which has been shown to play important roles in regulating metastasis and tumor progression [9]. Ovarian cancer cells with low E-cadherin levels are more invasive, and loss of E-cadherin expression in tumors is associated with poor survival [10, 11]. Our previous studies have demonstrated that activation of EGFR by different ligands contributes to the down-regulation of E-cadherin and increases the migration and invasion of human ovarian cancer cells [12–20].

Sprouty (SPRY) protein was first identified in *Drosophila* as an antagonist of fibroblast growth factor (FGF) in tracheal development [21]. Thus far, four *SPRY* genes (*SPRY1-4*) with sequence similarity to *Drosophila SPRY* have been identified in mammals [22]. Similar to *Drosophila* SPRY, mammalian SPRY inhibits the activation of ERK1/2 signaling by various growth factors [23]. SPRY is aberrantly expressed in different types of human cancer and is involved in tumorigenesis [24]. Our previous study and others' studies have shown that SPRY2 expression is down-regulated in human ovarian cancer and that patients with low SPRY2 expression have significantly poorer survival than those with high SPRY2 expression [25–28]. These results suggest that SPRY2 acts as a tumor suppressor in ovarian cancer progression. However, the mechanisms involved in SPRY2-mediated tumor suppression remain unknown and require further investigation.

Despite the importance of AREG and EGFR in ovarian tumorigenesis, whether SPRY2 is regulated by AREG and whether SPRY2 is involved in ovarian cancer progression are still unknown. The current study showed that treatment with AREG up-regulated SPRY2 expression in two human ovarian cancer cell lines, SKOV3 and OVCAR5. The stimulatory effect of AREG on SPRY2 expression was completely blocked by pre-treatment with an EGFR inhibitor, AG1478, and by siRNA-mediated EGFR knockdown. In addition, the effect of AREG on SPRY2 expression was abolished by inhibiting activation of the ERK1/2, but not the PI3K/AKT, signaling pathway. Moreover, we showed that overexpression of SPRY2 attenuated the AREG-induced down-regulation of E-cadherin, cell invasion and proliferation.

## RESULTS

### High AREG mRNA levels are associated with reduced disease-free survival in patients with ovarian cancer

**Figure 1:**
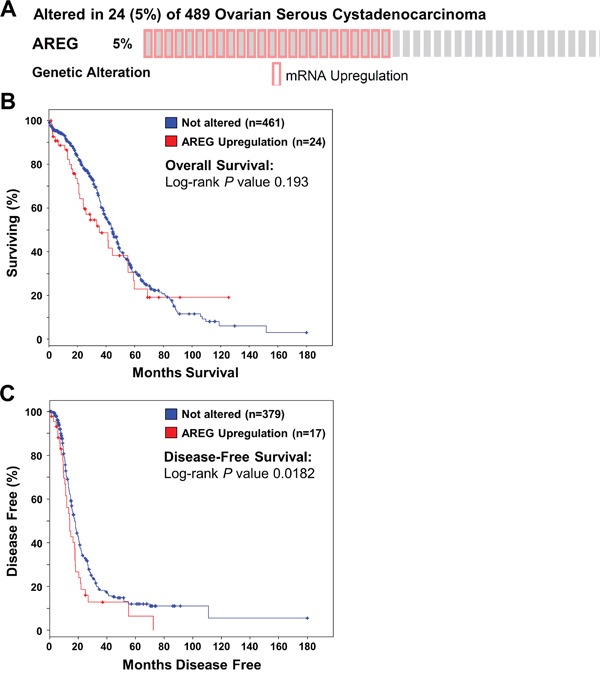
Up-regulation of AREG mRNA is associated with reduced disease-free survival in patients with high-grade serous ovarian carcinoma The cBioPortal for Cancer Genomics was used to query high-grade serous ovarian carcinomas from The Cancer Genome Atlas (n=489) for up-regulation of AREG mRNA above the median. **A.** OncoPrint results of cases with elevated AREG mRNA across all 489 high-grade serous ovarian carcinomas. **B** and **C.** Overall (B) and disease-free (C) survival differences between samples without and with elevated AREG mRNA levels were displayed as Kaplan-Meier survival curves with the associated *P* value (Log-rank test).

To investigate AREG expression and its relationship with ovarian cancer patient survival, we queried mRNA expression data on 489 high-grade serous ovarian carcinomas samples that were published by the Cancer Genome Atlas (TCGA) Research Network [29]. Using cBioPortal for Cancer Genomics [30], our analyses showed up-regulation of AREG mRNA in 24 (5%) of 489 cases (Figure 1A). No down-regulation of AREG mRNA was detected. Kaplan-Meier analyses showed that up-regulation of AREG mRNA was not significantly associated with overall survival (Log-rank *P* = 0.193) (Figure 1B). However, patients with up-regulated AREG mRNA had a significantly worse disease-free survival (Log-rank *P* = 0.0182) (Figure 1C). These results, together with the findings of a recent study, indicate that AREG contributes to tumor progression and poor survival in human ovarian cancer [7].

### AREG up-regulates SPRY2 by activating EGFR in human ovarian cancer cells

**Figure 2:**
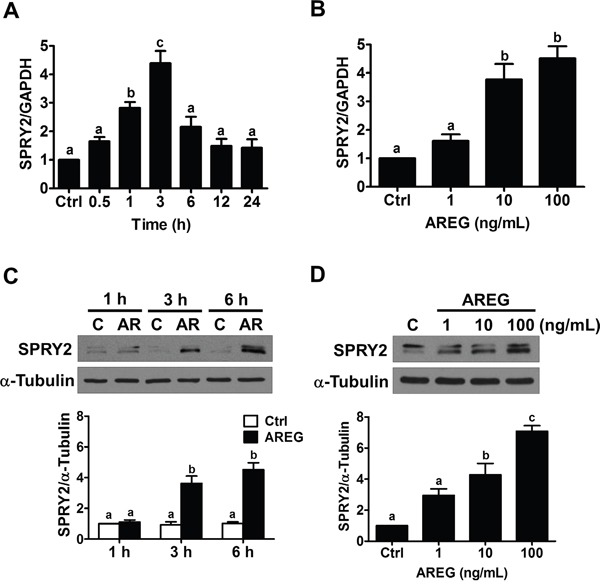
AREG up-regulates SPRY2 expression in human ovarian cancer cells **A.** SKOV3 cells were treated with 100 ng/mL AREG for different time periods, and SPRY2 mRNA levels were examined by RT-qPCR. SPRY2 mRNA level at each time point was normalized to GAPDH mRNA level at the same time point. **B.** SKOV3 cells were treated for 3 h with vehicle control or different concentrations of AREG, and SPRY2 mRNA levels were examined by RT-qPCR. **C.** SKOV3 cells were treated with 100 ng/mL AREG (AR) for different time periods, and SPRY2 protein levels were examined by Western blot. **D.** SKOV3 cells were treated for 3 h with vehicle control or different concentrations of AREG, and SPRY2 protein levels were examined by Western blot. The results are presented as the mean ± SEM of at least three independent experiments. Values without a common letter are significantly different (*P* < 0.05).

To examine the effect of AREG, two human ovarian cancer cell lines (SKOV3 and OVCAR5) that express AREG and EGFR were used as *in vitro* models [7, 19]. AREG protein levels can reach 100 ng/mL in normal human follicular fluid [31]. Therefore, we first treated SKOV3 cells with 100 ng/mL AREG for various periods of time. As shown in Figure 2A, 1 h treatment with AREG significantly up-regulated SPRY2 mRNA levels. The maximal effect was observed after 3 h of AREG treatment, and the induced AREG mRNA levels declined after 6 h of AREG treatment. We also examined the effect of different concentrations of AREG on SPRY2 expression. RT-qPCR results showed that treatment with 1 ng/mL AREG did not affect the mRNA levels of SPRY2 in SKOV3 cells, whereas treatment with 10 or 100 ng/mL AREG similarly and significantly up-regulated SPRY2 mRNA levels (Figure 2B). Western blot results showed similar effects: treatment with AREG transiently up-regulated SPRY2 protein levels in a dose-dependent manner (Figure 2C and 2D). Therefore, we used 10 ng/mL AREG for the following experiments.

**Figure 3:**
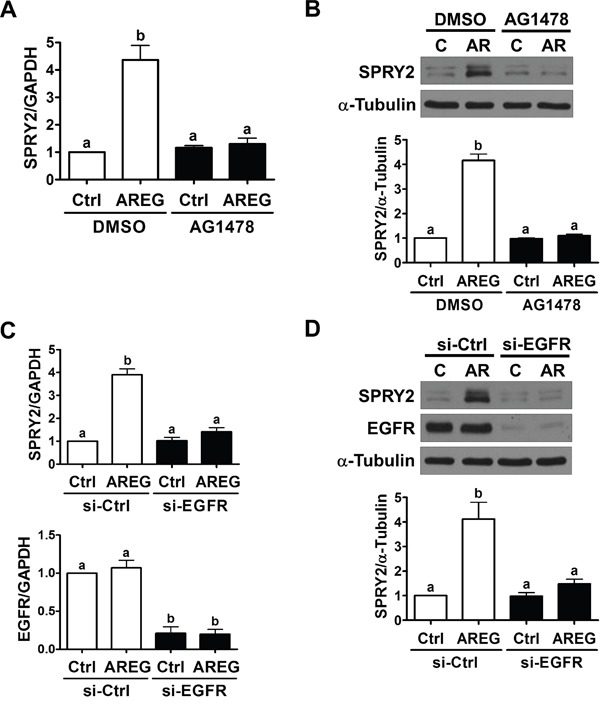
EGFR is required for the AREG-induced up-regulation of SPRY2 expression **A** and **B.** SKOV3 cells were pre-treated with vehicle control (DMSO) or 10 μM AG1478 for 1 h and then treated with vehicle control (Ctrl) or 10 ng/mL AREG (AR) for 3 h. The mRNA (A) and protein (B) levels of SPRY2 were examined by RT-qPCR and Western blot, respectively. **C** and **D.** SKOV3 cells were transfected with 50 nM control siRNA (si-Ctrl) or EGFR siRNA (si-EGFR) for 48 h and then treated with vehicle control (Ctrl) or 10 ng/mL AREG (AR) for 3 h. The mRNA (A) and protein (B) levels of SPRY2 and EGFR were examined by RT-qPCR and Western blot, respectively. The results are presented as the mean ± SEM of at least three independent experiments. Values without a common letter are significantly different (*P* < 0.05).

**Figure 4:**
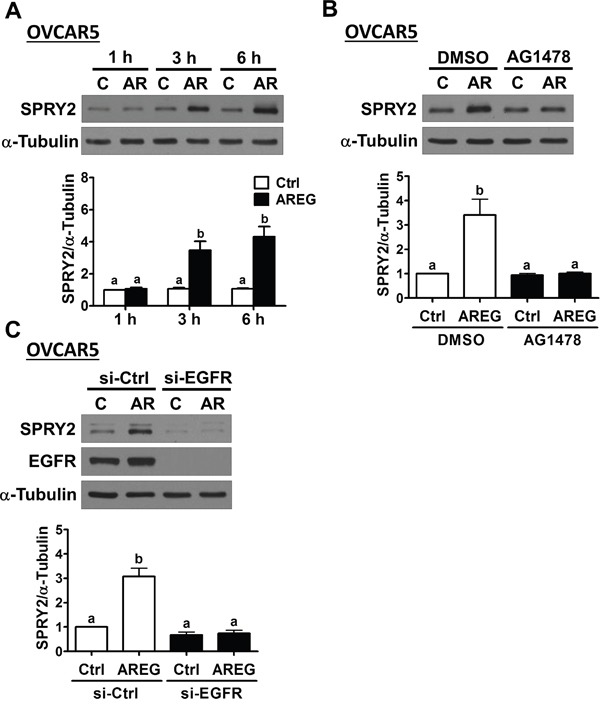
AREG up-regulates SPRY2 expression through EGFR in another human ovarian cancer cell line **A.** OVCAR5 cells were treated with 10 ng/mL AREG (AR) for different time periods, and SPRY2 protein levels were examined by Western blot. **B.** OVCAR5 cells were pre-treated with vehicle control (DMSO) or 10 μM AG1478 for 1 h and then treated with vehicle control (Ctrl) or 10 ng/mL AREG (AR) for 3 h. SPRY2 protein levels were examined by Western blot. **C.** OVCAR5 cells were transfected with 50 nM control siRNA (si-Ctrl) or EGFR siRNA (si-EGFR) for 48 h and then treated with vehicle control (Ctrl) or 10 ng/mL AREG (AR) for 3 h. The protein levels of SPRY2 and EGFR were examined by Western blot. The results are presented as the mean ± SEM of at least three independent experiments. Values without a common letter are significantly different (*P* < 0.05).

To confirm the requirement of EGFR in AREG-induced up-regulation SPRY2 expression, SKOV3 cells were pre-treated with an EGFR inhibitor, AG1478, to block the activation of EGFR. As shown in Figure 3A and 3B, pre-treatment with AG1478 completed blocked the stimulatory effects of AREG on SPRY2 mRNA and protein levels. To avoid off-target effects of the pharmacological inhibitor and further confirm that EGFR is required for AREG-induced SPRY2 expression, an siRNA-mediated knockdown approach was used to down-regulate endogenous EGFR expression. As shown in Figure 3C and 3D, EGFR siRNA significantly down-regulated EGFR mRNA and protein levels. In addition, the AREG-induced up-regulations of SPRY2 mRNA and protein levels were abolished by EGFR knockdown. AREG treatment similarly up-regulated SPRY2 expression in another human ovarian cancer cell line, OVCAR5 (Figure 4A). The stimulatory effect of AREG on SPRY2 expression in OVCAR5 cells was also abolished by inhibiting EGFR activity or expression (Figure 4B and 4C).

### ERK1/2, but not PI3K/AKT, signaling is involved in AREG-induced SPRY2 up-regulation

**Figure 5:**
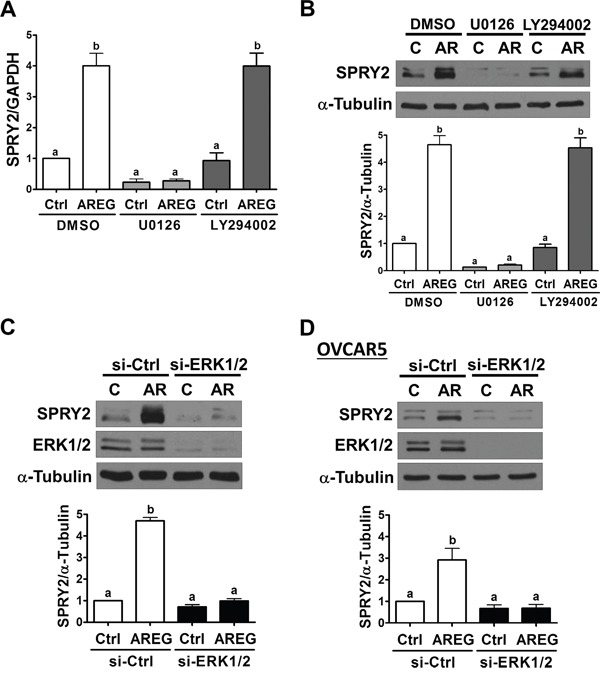
ERK1/2 is required for the AREG-induced up-regulation of SPRY2 **A** and **B.** SKOV3 cells were pre-treated with vehicle control (DMSO), 10 μM U0126 or 10 μM LY294002 for 1 h and then treated with vehicle control (Ctrl) or 10 ng/mL AREG (AR) for 3 h. The mRNA (A) and protein (B) levels of SPRY2 were examined by RT-qPCR and Western blot, respectively. **C** and **D.** SKOV3 (C) and OVCAR5 (D) cells were transfected with 50 nM control siRNA (si-Ctrl) or ERK1/2 siRNAs (si-ERK1/2) for 48 h and then treated with vehicle control (Ctrl) or 10 ng/mL AREG (AR) for 3 h. The protein levels of SPRY2 and ERK1/2 were examined by Western blot. The results are presented as the mean ± SEM of at least three independent experiments. Values without a common letter are significantly different (*P* < 0.05).

We have previously shown that AREG can activate the ERK1/2 and PI3K/AKT signaling pathways in human ovarian cancer cells [13]. Therefore, we next examined whether activation of the ERK1/2 or PI3K/AKT signaling pathway is required for SPRY2 up-regulation induced by AREG. RT-qPCR and Western blot results showed that pre-treatment with a MEK inhibitor, U0126, not only decreased basal SPRY2 mRNA and protein expression but also abolished the AREG-induced up-regulation of SPRY2 mRNA and protein levels in SKOV3 cells. Pre-treatment with a PI3K inhibitor, LY294002, did not affect basal or AREG-up-regulated SPRY2 expression (Figure 5A and 5B). To further confirm the involvement of ERK1/2 signaling in AREG-induced SPRY2 expression, ERK1/2 siRNAs were used to knockdown endogenous ERK1/2 expressions. As shown in Figure 5C, knockdown of ERK1/2 abolished the stimulatory effect of AREG on SPRY2 expression in SKOV3 cells. Similar results were observed in OVCAR5 cells (Figure 5D).

### Overexpression of SPRY2 attenuates AREG-induced down-regulation of E-cadherin expression

**Figure 6:**
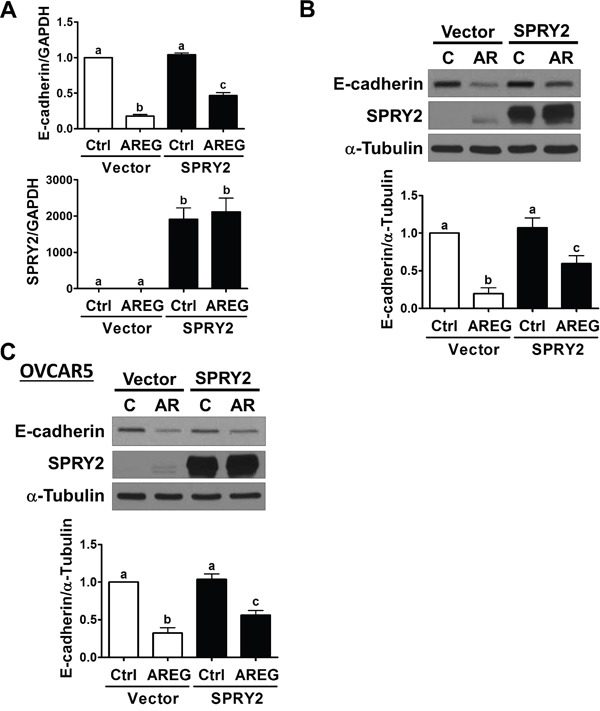
Overexpression of SPRY2 attenuates the AREG-induced down-regulation of E-cadherin expression **A** and **B.** SKOV3 cells were transfected for 48 h with 1 μg control vector (Vector) or SPRY2 overexpression vector (SPRY2) and then treated with 10 ng/mL AREG (AR) for 3 h. The mRNA (A) and protein (B) levels of E-cadherin and SPRY2 were examined by RT-qPCR and Western blot, respectively. **C.** OVCAR5 cells were transfected for 48 h with 1 μg control vector (Vector) or SPRY2 overexpression vector (SPRY2) and then treated with 10 ng/mL AREG (AR) for 3 h. The protein levels of E-cadherin and SPRY2 were examined by Western blot. The results are presented as the mean ± SEM of at least three independent experiments. Values without a common letter are significantly different (*P* < 0.05).

Our previous studies have shown that AREG induces human ovarian cancer cell invasion by down-regulating E-cadherin expression [13, 14]. Since SPRY2 expression is down-regulated in human ovarian cancer, we examined whether AREG-induced E-cadherin down-regulation can be attenuated by overexpressing SPRY2. As shown in Figure 6A and 6B, SKOV3 cells transfected with a vector encoding human SPRY2 expressed more SPRY2, whereas cells transfected with an empty vector did not show an up-regulation of SPRY2. Importantly, SPRY2 overexpression attenuated the AREG-induced down-regulation of E-cadherin mRNA and protein expression. Similarly, the inhibitory effect of AREG on E-cadherin expression was attenuated by SPRY2 overexpression in OVCAR5 cells (Figure 6C).

### Overexpression of SPRY2 attenuates AREG-induced Snail expression

**Figure 7:**
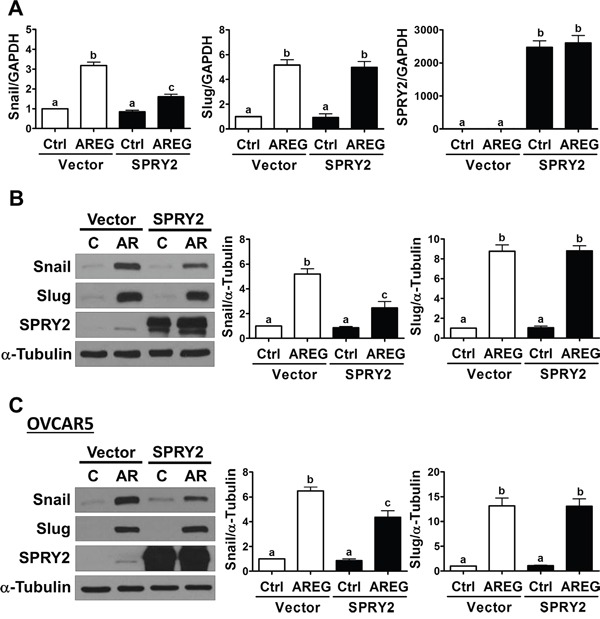
Overexpression of SPRY2 attenuates the AREG-induced up-regulation of Snail expression **A** and **B.** SKOV3 cells were transfected for 48 h with 1 μg control vector (Vector) or SPRY2 overexpression vector (SPRY2) and then treated with 10 ng/mL AREG (AR) for 3 h. The mRNA (A) and protein (B) levels of Snail, Slug and SPRY2 were examined by RT-qPCR and Western blot, respectively. **C.** OVCAR5 cells were transfected for 48 h with 1 μg control vector (Vector) or SPRY2 overexpression vector (SPRY2) and then treated with 10 ng/mL AREG (AR) for 3 h. The protein levels of Snail, Slug and SPRY2 were examined by Western blot. The results are presented as the mean ± SEM of at least three independent experiments. Values without a common letter are significantly different (*P* < 0.05).

It has been well characterized that E-cadherin can be down-regulated by increasing the expression of its transcriptional repressors, Snail and Slug [32]. Therefore, we examined whether SPRY2 overexpression affects the AREG-induced up-regulation of Snail and Slug. As shown in Figure 7A and 7B, AREG-treated SKOV3 cells showed increased Snail and Slug mRNA and protein levels. Interestingly, SPRY2 overexpression attenuated the AREG-induced up-regulation of Snail mRNA and protein levels without affecting basal Snail expression. However, SPRY2 overexpression did not affect the stimulatory effect of AREG on Slug expression. In OVCAR5 cells, overexpression of SPRY2 similarly attenuated the AREG-induced up-regulation of Snail, but not Slug, expression (Figure 7C).

### Overexpression of SPRY2 attenuates AREG-induced cell invasion and proliferation

**Figure 8:**
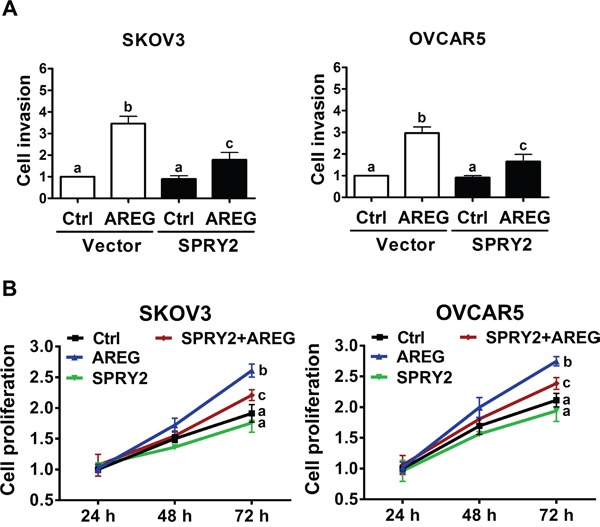
Overexpression of SPRY2 attenuates AREG-induced cell invasion and proliferation **A.** SKOV3 (left panel) and OVCAR5 (right panel) cells were transfected with 1 μg control vector (Vector) or SPRY2 overexpression vector (SPRY2) for 48 h. After transfection, the cells were treated with 10 ng/mL AREG, and the effects of AREG on cell invasion were examined in Matrigel invasion assays. **B.** SKOV3 (left panel) and OVCAR5 (right panel) cells were transfected with 1 μg control vector or SPRY2 overexpression vector (SPRY2) for 48 h. After transfection, the cells were treated with 10 ng/mL AREG every 24 h, and the number of cells was quantified using a trypan blue exclusion assay. The results are presented as the mean ± SEM of at least three independent experiments. Values without a common letter are significantly different (*P* < 0.05).

Given that overexpression of SPRY2 attenuated the AREG-induced down-regulation of E-cadherin in SKOV3 and OVCAR5 cells, we next examined whether SPRY2 overexpression can attenuate AREG-induced cell invasion. Matrigel invasion assays showed that treatment with AREG for 48 h increased the invasiveness of both SKOV3 and OVCAR5 cells. Importantly, overexpression of SPRY2 did not affect basal cell invasion but did significantly attenuate the AREG-induced invasion of both cell lines (Figure 8A). To confirm that the stimulatory effect of AREG on cell invasion was not due to the effects of AREG-induced cell growth, cell proliferation after AREG treatment was examined using a trypan blue exclusion assay. As shown in Figure 8B,

AREG treatment only increased cell proliferation after 72 h in culture. Interestingly, in both SKOV3 and OVCAR5 cells, overexpression of SPRY2 attenuated AREG-stimulated cell proliferation but did not significantly affect the basal cell proliferation.

### Down-regulation of SPRY2 mRNA is associated with reduced overall survival and disease-free survival in patients with ovarian cancer

**Figure 9:**
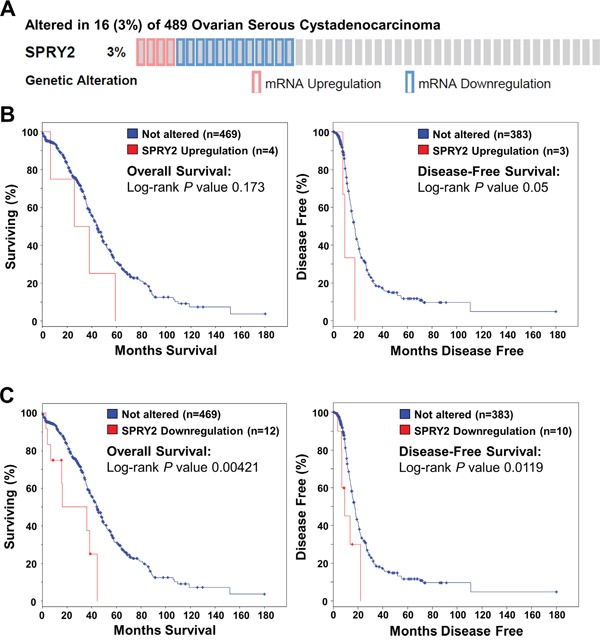
Down-regulation of SPRY2 mRNA is associated with reduced overall and disease-free survival in patients with high-grade serous ovarian carcinoma The cBioPortal for Cancer Genomics was used to query high-grade serous ovarian carcinomas from The Cancer Genome Atlas (n=489) for patients with altered SPRY2 mRNA levels. **A.** OncoPrint results showing cases with altered SPRY2 mRNA levels across all 489 high-grade serous ovarian carcinomas. **B.** Overall (left panel) and disease-free (right panel) survival differences between samples without and with up-regulated SPRY2 mRNA were displayed as Kaplan-Meier survival curves with the associated *P* value (Log-rank test). **C.** Overall (left panel) and disease-free (right panel) survival differences between samples without and with down-regulated SPRY2 mRNA were displayed as Kaplan-Meier survival curves with the associated *P* value (Log-rank test).

To examine the relationship between SPRY2 expression and the survival of patients with ovarian cancer, we queried the Cancer Genome Atlas (TCGA) Research Network dataset. As shown in Figure 9A, 16 (3%) of 489 cases had either up-regulation or down-regulation of SPRY2 mRNA levels. Up-regulation of SPRY2 mRNA was only detected in 4 (0.8%) of 489 cases, whereas 12 (2.5%) of 489 cases had down-regulation of SPRY2 mRNA. Kaplan-Meier analyses showed that up-regulation of SPRY2 mRNA was not associated with overall survival (Log-rank *P* = 0.173) or disease-free survival (Log-rank *P* = 0.05) (Figure 9B). Interestingly, patients with down-regulation of SPRY2 mRNA had significantly poorer overall survival (Log-rank *P* = 0.00421) and disease-free survival (Log-rank *P* = 0.0119) (Figure 9C). These results indicate that SPRY2 acts as a tumor suppressor in human ovarian cancer.

## DISCUSSION

SPRY2 expression levels are down-regulated in human ovarian cancer, and patients with low SPRY2 expression have significantly poorer survival than those with high SPRY2 expression [25–28]. In the present study, we provide new evidence that SPRY2 can antagonize the AREG-induced down-regulation of E-cadherin and invasion of human ovarian cancer cells. EGFR signaling not only regulates normal biological functions such as cell proliferation, migration, invasion and differentiation but also plays important roles in regulating tumorigenesis [33, 34]. Therefore, the down-regulation of SPRY2 in ovarian cancer decreases its ability to inhibit EGFR-mediated cellular functions, which subsequently contributes to tumor progression.

Accumulating evidence suggests that changes in the levels of local hormones and growth factors, along with their receptors and intracellular effectors, contribute to ovarian tumorigenesis [35, 36]. Multiple cognate ligands, including EGF, AREG, TGF-α, heparin-binding EGF (HB-EGF), epiregulin and betacellulin, can bind to and activate EGFR. However, only EGF, AREG and TGF-α bind EGFR exclusively; other ligands also bind other ERBB family receptors [3, 37, 38]. These EGFR ligands can be subdivided on the basis of their affinity for EGFR. EGF and TGF-α are considered high-affinity ligands while AREG is considered a low-affinity ligand [39, 40]. Previous studies have shown that AREG expression levels are significantly higher than EGF and TGF-α levels in ovarian cancer cells, tumor tissues and peritoneal and ascites fluid from patients with ovarian cancer [7, 41–43]. In addition, the current study and our previous studies show that AREG can stimulate ovarian cancer cell invasion by down-regulating E-cadherin expression [13, 14, 20]. Moreover, AREG has been reported to regulate ovarian cancer progression in an autocrine/paracrine manner [13, 44]. Overall, these studies indicate that AREG is the dominant EGFR ligand in ovarian cancer cells and plays important roles in promoting cancer development and progression. Indeed, a recent study demonstrates that shRNA-mediated AREG depletion or treatment with an anti-AREG monoclonal antibody inhibits the growth of human ovarian cancer cells [7]. Interestingly, cisplatin treatment increases AREG promoter activity but not EGF or TGF-α promoter activity, suggesting a possible role of AREG in regulating chemoresistance in ovarian cancer [7]. Taken together, these results suggest that targeting AREG is a potential new clinical approach that could lower the overall mortality and morbidity of human ovarian cancer.

It is interesting to note that despite the fact that multiple ligands can bind to and activate EGFR, they are capable of inducing different biological effects, even within the same cell [45]. For example, AREG, but not TGF-α, induces a morphological change and E-cadherin redistribution in MDCK cells [46]. In addition, AREG exhibits greater effect than EGF on cell motility and invasiveness of human mammary epithelial cells [5]. We have shown that treatment with EGF up-regulates SPRY2 expression, and overexpression of SPRY2 attenuates EGF-induced E-cadherin down-regulation and cell invasion in human ovarian cancer cells [25]. Given the evidence that AREG plays more important roles than EGF in the regulation of ovarian cancer progression, it is interesting and important to examine whether SPRY2 can be regulated by AREG and whether SPRY2 also affects the biological functions of AREG. The data shown in the present study demonstrated that overexpression of SPRY2 attenuated AREG-induced down-regulation of E-cadherin and invasion of human ovarian cancer cells. These results indicate that AREG and EGF induce similar biological functions in human ovarian cancer cells. In addition, in response to the AREG and EGF treatments, SPRY2 inhibits both EGFR ligands-induced E-cadherin down-regulation and cell invasion.

Since the discovery that SPRY proteins inhibit FGF receptor signaling during tracheal development in *Drosophila*, increasing evidence has characterized the critical role of SPRY proteins in regulating receptor tyrosine kinase-mediated MAPK/ERK1/2 signaling pathway [47]. Given their critical role as modulators of MAPK/ERK1/2 signaling, the SPRY proteins are expected to be deregulated in cancer. Thus far, aberrant expression of SPRY proteins has been identified in different types of human cancer [24]. Interestingly, in a context- and/or tissue-dependent manner, SPRY2 has been shown to act as a tumor suppressor [48–53] or promoter [54–57]. A recent study shows that SPRY2 is down-regulated in human ovarian cancer and that SPRY2 expression levels are negatively correlated with cell proliferation. Importantly, patients with lower tumor SPRY2 expression have significantly poorer overall survival and disease-free survival than those with high SPRY2 expression [28]. These results are consistent with our analyses of the TCGA dataset in the present study. In addition, we showed that overexpression of SPRY2 attenuated AREG-induced ovarian cancer cell invasion. These results indicate that SPRY2 may act as a tumor suppressor in human ovarian cancer and could be used as a prognostic biomarker. Although it is not presently possible to affect SPRY2 function with small molecules, efforts to increase the specificity and efficiency of viral vectors or non-viral vectors to modulate gene expression are in ongoing development [58, 59]. Therefore, through the more thorough dissection of the targets or signaling pathways that are regulated by SPRY2 could lead to the development of alternative therapeutic approaches for human ovarian cancer.

In the mouse, SPRY1, SPRY2 and SPRY4 expression can be detected in various embryonic tissues, whereas SPRY3 is only expressed in the adult brain and testis [22, 60]. In ovarian cancer, SPRY4 expression levels are also down-regulated, while the changes in SPRY1 expression remain controversial [25, 27, 28, 61]. Our recent study demonstrates that SPRY4 expression is involved in the AREG-induced down-regulation of E-cadherin and invasion of human ovarian cancer cells [20]. However, in the present study, we showed that SPRY2 antagonized the AREG-induced down-regulation of E-cadherin and cell invasion. These results indicate that SPRY2 and SPRY4 have opposing effects on the regulation of EGFR-mediated E-cadherin down-regulation and cell invasion in human ovarian cancer. Although SPRY4 down-regulation is detected in ovarian cancer, the expression levels of SPRY4 do not significantly correlate with overall survival or disease-free survival [28]. These results suggest that SPRY2 plays a more important role than SPRY4 in regulating human ovarian cancer progression. Future studies will be needed to investigate the molecular mechanisms underlying the different roles of SPRY2 and SPRY4 in the regulation of EGFR-mediated cellular function in human ovarian cancer.

We have shown that AREG up-regulates Snail and Slug expression, and both Snail and Slug are required for the AREG-induced down-regulation of E-cadherin in human ovarian cancer cells [13, 14]. The results of the present study showed that basal Snail, Slug and E-cadherin expression was not affected by SPRY2 overexpression in two human ovarian cancer cell lines, SKOV3 and OVCAR5, while the AREG-induced up-regulation of Snail, down-regulation of E-cadherin and increase in cell invasion were attenuated by SPRY2 overexpression. Interestingly, in contrast to our results, SPRY2 can suppress both basal and 1α,25-dihydroxyvitamin D3-induced E-cadherin expression, and SPRY2 expression levels are negatively correlated with E-cadherin expression levels in human colon cancer cells [54]. These results suggest cancer type-specific roles of SPRY2 in cancer development and progression.

In summary, the present study demonstrates that AREG expression levels are negatively correlated with patient survival. Treatment with AREG up-regulates SPRY2 expression in two human ovarian cancer cell lines. The stimulatory effect of AREG on SPRY2 expression is mediating by the ERK1/2 signaling pathway. In addition, our results show that overexpression of SPRY2 attenuates the AREG-induced up-regulation of Snail, down-regulation of E-cadherin, cell invasion and proliferation. Moreover, down-regulation of SPRY2 expression is associated with poor survival. These results suggest a tumor suppressor role for SPRY2 in human ovarian cancer and provide a possible target for the development of novel therapeutic strategies.

## MATERIALS AND METHODS

### Cell culture

The SKOV3 human ovarian cancer cell line was obtained from American Type Culture Collection (Manassas, VA). The OVCAR5 human ovarian cancer line was kindly provided by Dr. T.C. Hamilton (Fox Chase Cancer Center, Philadelphia, PA). Cells were grown in a 1:1 (v/v) mixture of M199/MCDB105 medium (Sigma, Oakville, ON) supplemented with 10% fetal bovine serum (FBS; HyClone Laboratories Inc., Logan, UT). The cultures were maintained at 37°C in a humidified 5% CO_2_ atmosphere.

### Antibodies and reagents

The polyclonal anti-Sprouty2 antibody (#S1444) was obtained from Sigma. The monoclonal anti-E-cadherin antibody (#610181) was obtained from BD Biosciences (Mississauga, ON). The polyclonal anti-EGFR (#sc-03) and monoclonal anti-α-tubulin (#sc-23948) antibodies were obtained from Santa Cruz Biotechnology (Santa Cruz, CA). The polyclonal anti-ERK1/2 (#9102), anti-Slug (#9585) and monoclonal anti-Snail (#3895) antibodies were obtained from Cell Signaling Technology (Danvers, MA). Horseradish peroxidase-conjugated goat anti-mouse IgG and goat anti-rabbit IgG were obtained from Bio-Rad Laboratories (Hercules, CA). AG1478 and LY294002 were obtained from Sigma. U0126 was obtained from Calbiochem (San Diego, CA). Recombinant human AREG was purchased from R&D Systems (Minneapolis, MN).

### Small interfering RNA (siRNA) transfection and overexpression

For endogenous EGFR or ERK1/2 knockdown, the cells were transfected with 50 nM ON-TARGET*plus* SMARTpool specific siRNA (Dharmacon Research, Inc., Lafayette, CO) using Lipofectamine RNAiMAX (Invitrogen, Burlington, ON). Non-targeting siCONTROL siRNA (Dharmacon) was used as a transfection control. To overexpress human SPRY2, the pXJ40-FLAG-SPRY2 vector and empty pXJ40-FLAG vector (gifts from Dr. Graeme R. Guy, Institute of Molecular and Cell Biology, Singapore) [62] were transfected using Lipofectamine 3000 (Invitrogen).

### Real-time quantitative PCR (RT-qPCR)

Total RNA was extracted using TRIzol reagent (Invitrogen) according to the manufacturer's instructions. Reverse transcription was performed with 3 μg RNA, random primers and M-MLV reverse transcriptase (Promega, Madison, WI). The primers used for SYBR Green RT-qPCR were as follows: SPRY2, forward 5′-CCC CTC TGT CCA GAT CCA TA-3′ and reverse 5′-CCC AAA TCT TCC TTG CTC AG-3′; EGFR, forward 5′-GGT GCA GGA GAG GAG AAC TGC-3′ and reverse 5′-GGT GGC ACC AAA GCT GTA TT-3′; E-cadherin, forward 5′-ACA GCC CCG CCT TAT GAT T-3′ and reverse 5′-TCG GAA CCG CTT CCT TCA-3′; Snail, forward 5′-CCC CAA TCG GAA GCC TAA CT-3′ and reverse 5′- GCT GGA AGG TAA ACT CTG GAT TAG A-3′; Slug, forward 5′-TTC GGA CCC ACA CAT TAC CT-3′ and reverse 5′-GCA GTG AGG GCA AGA AAA AG-3′; and GAPDH, forward 5′-GAG TCA ACG GAT TTG GTC GT-3′ and reverse 5′-GAC AAG CTT CCC GTT CTC AG-3′. RT-qPCR was performed using an Applied Biosystems 7300 Real-Time PCR System (Perkin-Elmer) equipped with a 96-well optical reaction plate. All RT-qPCR data are presented as the mean of at least three independent experiments conducted in triplicate. The relative quantification of mRNA levels was performed by the comparative Ct method using GAPDH as the reference gene and the formula 2^-ΔΔCt^.

### Western blot

Cells were lysed in lysis buffer (Cell Signaling Technology), and protein concentration was determined using a DC protein assay kit with BSA as the standard (Bio-Rad Laboratories). Equal amounts of protein were separated by SDS-polyacrylamide gel electrophoresis and transferred to PVDF membranes. After blocking in Tris-buffered saline (TBS) containing 5% non-fat dry milk for 1 h, the membranes were incubated overnight at 4°C with primary antibodies, followed by incubation with the HRP-conjugated secondary antibody. Immunoreactive bands were detected with an enhanced chemiluminescent substrate (Pierce, Rockford, IL).

### Invasion assay

Invasion assays were performed in Boyden chambers with minor modifications [63]. Cell culture inserts (24-well, 8-μm pore size; BD Biosciences, Mississauga, ON) pre-coated with growth factor-reduced Matrigel (40 μL, 1 mg/mL; BD Biosciences) were used for invasion assays. Cell culture inserts were seeded with 1x10^5^ cells in 250 μL of medium with 0.1% FBS. Medium with 10% FBS (750 μL) was added to the lower chamber and served as a chemotactic agent. After 48 h incubation, non-invading cells were removed from the upper side of the membrane, and cells on the lower side were fixed with cold methanol and air dried. The cells were stained with crystal violet (Sigma) and counted. Each individual experiment was performed with triplicate inserts, and five microscopic fields were counted per insert.

### Statistical analysis

The results are presented as the mean ± SEM of at least three independent experiments. The results were analyzed by one-way ANOVA and Tukey's multiple comparison test using PRISM software. Significant differences were defined by values of *P* < 0.05.
